# Variability in sulfur isotope composition suggests unique dimethylsulfoniopropionate cycling and microalgae metabolism in Antarctic sea ice

**DOI:** 10.1038/s42003-018-0228-y

**Published:** 2018-12-04

**Authors:** Gauthier Carnat, Ward Said-Ahmad, François Fripiat, Boris Wittek, Jean-Louis Tison, Christiane Uhlig, Alon Amrani

**Affiliations:** 1 Laboratoire de Glaciologie, Université Libre de Bruxelles, Brussels, B-1050 Belgium; 2 Institute of Earth Science, The Hebrew University of Jerusalem, Jerusalem, 91904 Israel; 3 Climate Geochemistry Department, Max Planck Institute for Chemistry, Mainz, 55128 Germany; 4 Section Marine Geochemistry, Alfred Wegener Institute, Helmholtz Centre for Polar and Marine Research, Bremerhaven, 27570 Germany

**Keywords:** Biogeochemistry, Metabolic pathways, Microbial ecology

## Abstract

Sea ice microbial communities produce large amounts of the sulfur metabolite dimethylsulfoniopropionate (DMSP), a precursor of the climate cooling gas dimethylsulfide. Despite their importance to the polar sulfur cycle, drivers and metabolic pathways of sea ice DMSP are uncertain. Here we report the first measurements of sea ice DMSP sulfur isotopic composition (^34^S/^32^S ratio, δ^34^S). δ^34^S values in ice cores from the Ross Sea and Weddell Sea reveal considerable variability across seasons and between ice horizons (from +10.6 to +23.6‰). We discuss how the most extreme δ^34^S values observed could be related to unique DMSP cycling in the seasonally extreme physiochemical conditions of isolated brine inclusions in winter-spring. Using cell cultures, we show that part of the DMSP δ^34^S variability could be explained by distinct DMSP metabolism in sea ice microalgae. These findings advance our understanding of the sea ice sulfur cycle and metabolic adaptations of microbes in extreme environments.

## Abstract (web summary)

Gauthier Carnat et al. report the first measurements of the natural sulfur isotopic composition of sea ice from the Antarctic. They find considerable variability between seasons and sea ice horizons, suggesting unique cycling of sulfur metabolites by sea ice microalgae in extreme environments, with important implications for the production of gasses that impact global climate.

## Introduction

Dimethylsulfoniopropionate (DMSP) is an important sulfur metabolite synthesized by marine microalgae^1^ and bacteria^2^. Multiple physiological roles have been suggested for DMSP to aid in environmental stress adaptation including cryoprotection^3^, osmoregulation^4^, and protection against oxidative stress^5^. DMSP is also a major precursor of the most abundant volatile sulfur compound in oceanic waters, dimethylsulfide (DMS). Oceanic emissions of DMS represent about 25% of the global sulfur flux to the atmosphere^6^, where it is quickly oxidized to condensable acidic sulfur species^7^. As a precursor of sunlight-scattering sulfate aerosols and cloud-condensation nuclei, DMS could play a role in climate warming mitigation, although the level of its contribution is still debated^8,9^.

DMS climate-cooling potential may prove to be particularly relevant in the climate-sensitive and aerosol-poor polar regions^10^. DMS emissions in these regions are strongly influenced by the development of seasonal sea ice. Liquid salty micro-inclusions (brine), which remain trapped between sea ice crystals on freezing, provide a habitat for metabolically active microbial communities (sympagic communities) which can support very high biomass^11^. These communities are known to periodically produce DMS and DMSP concentrations that are several orders of magnitude greater than global oceanic means^3,12,13^, and release of these sulfur and microbial pools at the ice edge during the spring–summer melt season have been correlated to strong DMS pulses and atmospheric aerosols formation events^14^.

Despite its potential importance for the sulfur cycle in the polar ocean and atmosphere, the biogeochemical cycling of sea ice DMS and DMSP is still poorly understood. Field measurements of sea ice DMS and DMSP concentrations show considerable spatial and temporal variability^13^ which is currently not well predicted by models. Some of this variability has been correlated with the seasonal evolution of sympagic algal biomass and its vertical zonation in taxonomically distinct assemblages^15–17^, DMSP production being known to differ strongly among taxonomic groups^18^. Sympagic microbial communities also thrive in seasonally and vertically variable physiochemical conditions, mainly driven by changes in sea ice thermodynamics and brine dynamics^19^. For instance, sympagic microbes must cope with salinities ranging from < 10 g kg^−1^ in summer melt channels to over 200 g kg^−1^ in isolated winter brine pockets, and with temperatures ranging from −20 °C in winter surface ice to −1.8 °C at the ice–ocean interface^11^. High variability in oxygen levels, nutrient supply, light availability, and pH between the different sea ice habitats have also been reported^20^. As suggested by many^3,4,21,22^, such variability in physiochemical conditions should strongly influence microbial processes driving the production and degradation of DMS and DMSP^1^. Unfortunately, only the influence of brine salinity and temperature on microalgal cell physiology and DMSP synthesis was investigated in some details^4,23^. Moreover, the exact contribution of sea ice DMS to the annual oceanic DMS flux to the atmosphere remains extremely difficult to quantify. Sea ice may transfer DMS to the polar ocean and ultimately to the atmosphere via multiple pathways such as brine drainage at the ice edge during the entire season^13,24^ and melting and ice-breakup in summer^14^. Direct emissions of DMS from the ice surface and leads have also been observed^25^.

Natural isotopes measurement is a powerful approach to trace sources and transformation processes in complex biogeochemical cycles^26^. A sensitive method for δ^34^S analysis in DMS and DMSP in seawater samples was developed in 2013, coupling gas chromatography (GC) and multicollector inductively coupled plasma mass spectrometry (MC-ICPMS)^27^. Using this method, Amrani et al.^28^ analysed surface water DMSP samples from six different ocean provinces, revealing a remarkable consistency in δ^34^S values. Other studies in non-oceanic aquatic environments with different physiochemical conditions such as salt marshes^28^ and freshwater lakes^29^ reported very different isotopic values. Until now, the natural sulfur isotopic composition (^34^S/^32^S ratio, δ^34^S) of DMSP had never been measured in the highly variable sea ice environment.

In this study, we present the first assessment of DMSP δ^34^S variability in Antarctic sea ice. Considerable variability is revealed across seasons and between sea ice horizons, with multiple values falling out of the range observed in oceanic waters. We show that the highest variability and most extreme DMSP δ^34^S values are generally found in cold and highly saline isolated brine pockets, while the isotopic signatures in warmer and fresher connected brine channels are more homogeneous. We discuss how the variability could relate to brine inclusions connectivity and its effect on mixing, and to the cycling of DMSP by bacteria and heterotrophs in the isolated brine pockets. Using cell cultures of the polar diatom *Fragilariopsis cylindrus* in brine conditions, we show that part of the variability could originate from distinct DMSP metabolism in sea ice algae.

## Results

### Overall variability of sea ice DMSP δ^34^S

**Fig. 1:**
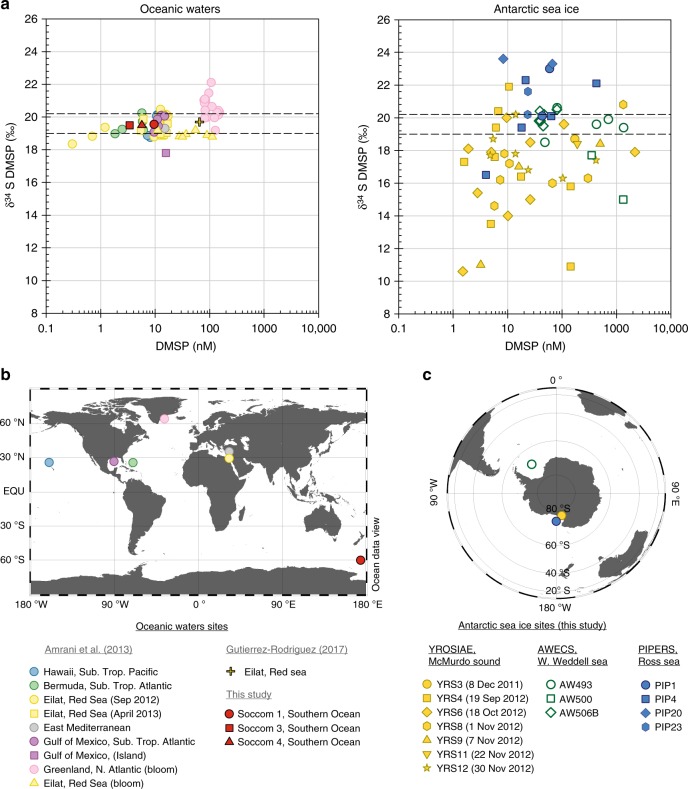
DMSP δ^34^S variability in oceanic waters and Antarctic sea ice. **a** Sulfur isotope ratio (δ^34^S, ‰) and concentrations (nanomolar) of dimethylsulfoniopropionate (DMSP) in seawater from different ocean basins, including the Southern Ocean (left) and in Antarctic sea ice samples collected in this study (right). Note the log scale for the DMSP concentrations. The area between the dashed lines indicates the range of δ^34^S values measured in oceanic surface waters only (≤5 m). **b** Location of the oceanic waters sampling sites available in the literature, including the Southern Ocean samples from this study. **c** Location of the sea ice sampling sites and stations analysed in this study

The variability of DMSP δ^34^S values in sea ice was assessed from a set of Antarctic sea ice cores collected in two different regions of the Southern Ocean (Western Weddell Sea and Ross Sea, see Fig. 1). Pack ice was sampled at three different stations in the Western Weddell Sea (AWECS field study^30^), and at four different stations in the central Ross Sea and Ross Sea marginal ice zone (PIPERS field study^31^). One land-fast ice station was visited seven times during a year-round time series study in the McMurdo Sound, Ross Sea (YROSIAE field study^13^). At each station, DMSP δ^34^S were determined as a function of depth, targeting distinct sea ice horizons (surface, interior, bottom ice, and ice–ocean interface) and their associated microalgal assemblages.

Overall, sea ice DMSP δ^34^S showed very high variability, with values ranging between +10.6 and +23.6‰ (*n* = 65) (Fig. 1a). To put this variability in perspective, all the oceanic DMSP δ^34^S reported in the literature fall within a much narrower range of +17.3 to +22.1‰^28,32,33^ (+18.9 to +20.3‰ for near-surface (0–5 m) waters) (Fig. 1a). The majority of sea ice DMSP δ^34^S values were comprised between +14 and +22‰, but a few measurements showed surprisingly light (between +10.6 and +12‰) δ^34^S values. Such light DMSP δ^34^S have been recorded in non-oceanic aquatic environments such as salt marshes (+11.3‰)^28^ and freshwater lakes (+11.4‰)^29^, where the source sulfate for DMSP metabolism was much lighter than seawater sulfate. Closer inspection of sea ice DMSP δ^34^S values reveals substantial variability between the three sets of ice cores collected (Fig. 1a). Pack ice from the Western Weddell Sea showed values ranging from +15 to +20.6‰, with a mean (+19.2‰ ± 1.6‰) within the near-surface oceanic waters range. Pack ice from the Ross Sea had mostly heavier values, ranging from +16.5 to +23.6‰ with a mean of +21.2‰ ± 1.7‰. The most striking variability was observed in land-fast ice from the McMurdo Sound with values ranging between very light (+10.6‰) and heavy (+21.9‰) δ^34^S with a standard deviation of ±2.6‰. The mean DMSP δ^34^S of this set of ice cores (+17.0‰) was also much lighter than in the two other sets. No correlation was found between DMSP δ^34^S and DMSP concentrations in any of the ice core sets (Fig. 1a, Supplementary figure 1, 2), as also reported in oceanic water samples^28^.

### DMSP δ^34^S variability between seasons and across ice horizons

**Fig. 2:**
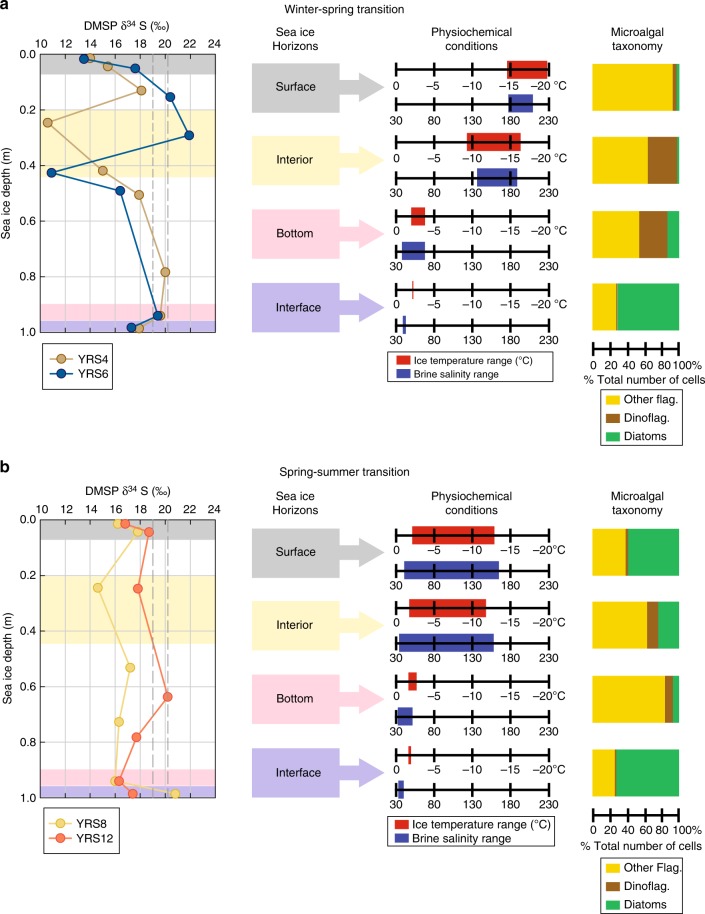
Differences in sea ice DMSP δ^34^S, physiochemical conditions and microalgal taxonomy between two distinct seasonal periods in the McMurdo Sound. **a** Depth profiles of DMSP δ^34^S values in sea ice from two stations in the McMurdo Sound sampled during the winter–spring transition (19 September–18 October 2012). δ^34^S values in four distinct sea ice horizons (surface ice, interior ice, bottom ice, and ice–ocean interface) are compared to the temperature and brine salinity range observed in these horizons during the transition, and the microalgal taxonomy observed in these horizons halfway through the transition (5 October 2012). **b** Similar comparison for two stations in the McMurdo Sound sampled during the spring–summer transition (1 November–30 November 2012). Sea ice depths were normalized. The vertical dashed lines represent the range of DMSP δ^34^S values observed in surface oceanic waters

The YROSIAE study in the McMurdo Sound provided an opportunity to sample the same fast-ice site from the winter–spring transition (mid-September) to the spring–summer transition (late-November), covering the full seasonal cycle (growth and decay) of a sympagic bloom^13^. This set of ice cores was therefore selected to study the temporal/seasonal variability in sea ice DMSP δ^34^S, since the two other sets integrated spatial variability and were sampled over a much shorter time frame^30,31^. Temporal trends during the YROSIAE study are described here in four distinct sea ice horizons corresponding to distinct microalgal assemblages (surface, interior, bottom ice, and ice–ocean interface)^13^ (Fig. 2a, b). Two contrasting seasonal periods (winter–spring transition vs. spring–summer transition) were targeted based on atmospheric conditions (air temperature and light), sea ice thermodynamics (ice temperature, brine salinity, permeability), and the state of sympagic communities (chl-*a* and phaeopigments) as described in ref. ^13^. While the mean DMSP δ^34^S of each station were relatively similar (ranging between +16.5 and +17.8‰, averaging +17.1‰ ± 0.5‰), sharp contrasts could be observed between the shape and range of the depth profiles (Fig. 2a, b). The winter–spring transition cores showed the most extreme light values in ^34^S (located in interior ice), and a strong vertical variability between the surface/interior ice horizons and bottom/ice–ocean interface horizons. This variability observed in <2 m of sea ice was higher than the overall variability observed in surface oceanic waters, and in 120 m depth seawater profiles from the Red Sea^28^. Interestingly, the late-winter core (YRS4) also had a heavy δ^34^S value in its interior ice horizon. On the other hand, the spring–summer transition cores showed more homogeneity. A trend of ^34^S depletion in surface ice compared to sub-surface ice was observed in all cores, and bottom/ice–ocean interface δ^34^S values were always very close to the typical oceanic waters range.

Available oceanic DMSP δ^34^S values sampled in six different ocean provinces encompass a wide range of hydrological, meteorological, and biological conditions^28,32^ (Fig. 1b). However, DMSP δ^34^S had never been measured in the Southern Ocean close to our sea ice sampling sites (Fig. 1c). We collected seawater samples at three SOCCOM (Southern Ocean Carbon and Climate Observations and Modelling project) float locations in the Ross Sea sector of the Southern Ocean (Fig. 1b). SOCCOM 1 was sampled at 60°S 170°E, SOCCOM 3 at 67.5°S 172°E, and SOCCOM 4 at 68.6°S 172°E, within a few nautical miles of the marginal ice zone (68.8°S). The three locations yielded very consistent DMSP δ^34^S values, averaging +19.5‰ (±0.2‰), which fall within the range of near-surface oceanic waters signatures (Fig. 1a).

### Sulfur isotope signature of sea ice SO_4_

The S source for microalgal DMSP synthesis in oxygenated parts of the oceanic water column is widely accepted to be dissolved sulfate (SO_4_^2−^), taken up through assimilatory sulfate reduction^1,34^. The concentration of SO_4_^2−^ in sea ice is regulated by a complex interplay between the concentration/dilution of salts as sea ice forms/melts, the temperature-driven sequential precipitation/dissolution of sulfate minerals (gypsum and mirabilite), and brine exchanges with seawater in the case of an open permeable brine system^35–37^. As a result, the bioavailability of sulfate is much more variable in the brine habitat than in oceanic waters which show a stable concentration of 28 mM^34^. Evaluating the SO_4_^2−^ concentration and δ^34^S signature in our sea ice sample was therefore of specific interest. Sulfate concentrations were determined in bulk ice from the YROSIAE study and weighted by the brine volume fraction to estimate the amount of sulfate available in brine (Supplementary figure 1). Concentrations were highly variable, ranging from 12.8 mM at the summer station YRS12 to 439.1 mM at the winter station YRS4, but always high enough to sustain sulfur metabolism^36^. SO_4_^2−^ has a very homogeneous δ^34^S in most oceanic waters (~+21‰, +21.3‰ ± 0.1‰ in under-ice water in the McMurdo Sound)^38,39^. Sulfur isotope measurements of bulk ice sulfate in the ice core set from the McMurdo Sound (Supplementary figure 1) strongly suggest this is also the case in sea ice. The δ^34^S values showed homogeneity across seasons and between sea ice horizons with an overall mean identical to the oceanic signature (+21.2‰ ± 0.2‰, *n* = 18). These results are consistent with those reported in frost flowers and sea ice in the Canadian High Arctic, and support the assumption of no isotopic fractionation during mirabilite formation^40^.

### Variability in DMSP δ^34^S in cell cultures experiments

**Fig. 3:**
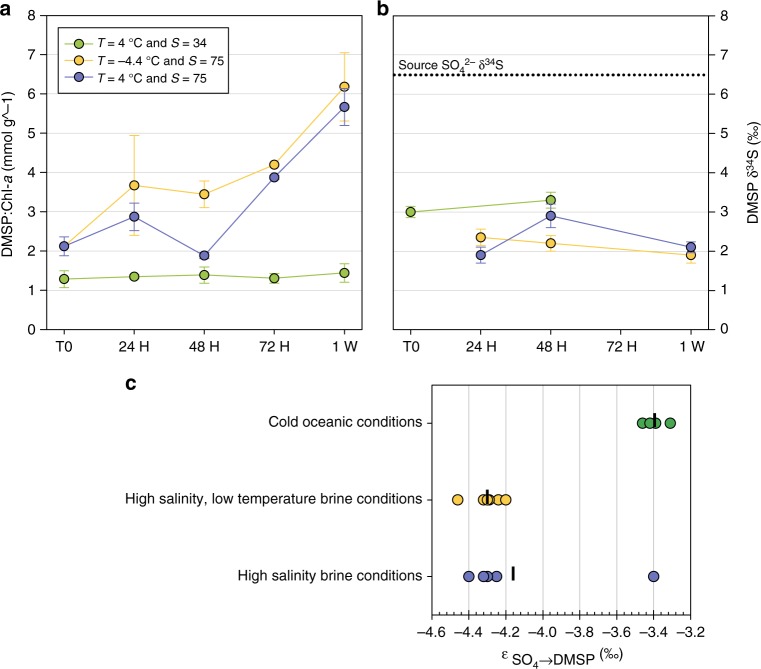
Changes in DMSP:Chl*a* and DMSP δ^34^S over time and fractionation factors between SO_4_ and DMSP in cultures of *F. cylindrus* in cold oceanic conditions and in brine conditions. **a** Changes in DMSP:Chl*a* (mmol/g) observed over a 1 week period in cell cultures of the polar diatom *F. cylindrus* in cold oceanic conditions and in two brine conditions (high salinity, low temperature; high salinity only). Error bars represent SD between triplicates. **b** Changes in DMSP δ^34^S in the same cultures over the same period. The horizontal dotted line represents the S isotopic value of the source sulfate in the water mix used for all the cultures. Error bars represent SD between duplicates. **c** Isotope differences (apparent fractionation *ε*) between SO_4_^2−^ and DMSP calculated for each culture. The vertical bars represent averages

In addition to field measurements, we conducted controlled experiments with sympagic microalgal cell cultures. We looked at the potential variability in δ^34^S of the DMSP production of axenic cultures of the diatom *F. cylindrus* following salinity (*S*) and temperature (*T*) shifts typically encountered by these organisms in the brine habitat across seasons^11,20^. *F. cylindrus* is an ice-associated diatom widespread in both polar regions whose cell physiology and DMSP metabolism in sea ice physiochemical conditions have been studied in some details^23^. Temperature and salinity are known not only to control DMSP production in ice-associated algae^3,13,23^ but also to control the abundance and activity of enzymes involved in microalgal sulfate assimilation^41^ and DMSP synthesis^21,42^. *F. cylindrus* cultures were brought from cold oceanic conditions (*T* = 4 °C, *S* = 34 g kg^−1^) to high salinity conditions (*T* = 4 °C, *S* = 75 g kg^−1^), and to combined high salinity–low temperature conditions (*T* = −4.4 °C, *S* = 75 g kg^−1^) (Fig. 3), and sampled several times over the course of a week. As expected, a net increase in DMSP:chl-*a* ratio (up to 600% after 1 week) was measured in both experiments (Fig. 3a). More interestingly, variability in DMSP δ^34^S was also observed between the control culture and the experiments (Fig. 3b). DMSP δ^34^S in the control culture (cold oceanic conditions) at the start of the experiment and after 48 h were relatively similar, averaging +3.1‰ ± 0.2‰ (*n* = 4). Considering that the source sulfate in the culture water (mix of natural seawater and artificial salts) had a δ^34^S of +6.5‰ (± 0.1‰), the corresponding apparent fractionation factor from sulfate to DMSP was on average −3.4‰ ± 0.06‰ (*n* = 4) (Fig. 3c). This value is at the lower end of assimilatory fractionations observed in oceanic microalgae^43^. DMSP δ^34^S in the high salinity–low temperature culture (*T* = −4.4 °C, *S* = 75 g kg^−1^) after 24 h, 48 h, and 1 week were also relatively similar (on average +2.1‰ ± 0.2‰, *n* = 6) but ±1‰ lighter than in the control culture, yielding an apparent fractionation factor of −4.3‰ ± 0.09‰ (*n* = 6) on average (Fig. 3c). DMSP δ^34^S in the high salinity culture (*T* = 4 °C, *S* = 75 g kg^−1^) were slightly more variable (Fig. 3b). δ^34^S after 24 h (+1.9‰) and 1 week (+2.1‰) were comparable to those of the high salinity–low temperature culture, but heavier after 48 h (+2.9‰), yielding an apparent fractionation factor of −4.2‰ ± 0.4‰ on average (*n* = 6) (Fig. 3c).

## Discussion

The apparent lack of correlation between DMSP concentrations and DMSP δ^34^S in all the ice core sets suggests that more than one process drive the isotopic signature of DMSP in sea ice. The results of the present study point toward an important role of sea ice thermodynamic growth and the vertical/seasonal development of the brine inclusions network. In particular, the stark contrast between small, cold, and highly saline isolated brine pockets that develop in cold ice, and larger, warmer, and fresher connected brine channels that develop in warmer ice warrants further consideration (Fig. 2a, b).

Sea ice variability in DMSP δ^34^S could partially be explained by differences in mixing processes driven by the connectivity state of the brine network. Mixing likely helps to homogenize the isotopic signature of DMSP in surface oceanic waters. In contrast, mixing was very likely inhibited in some of our sea ice samples. Mixing in sea ice is inhibited when the ice temperature/salinity-dependent brine volume fraction drops below a ~5% threshold^44^. Brine inclusions become isolated from each other, drastically reducing the fluid permeability of sea ice^16,19,20,45^. Brine volume fractions <5% were observed in the cold surface and interior ice horizons of the winter–spring stations 4 and 6 and spring–summer station 8 in the McMurdo Sound ice core set (Supplementary figure 3). Most of the extreme DMSP δ^34^S values presented in this study were recorded in these horizons (Fig. 2 and Supplementary figure 4). In the warmer bottom/ice–ocean interface horizons and in the warmer summer station 12, brine volume fractions largely exceeded the permeability threshold (Supplementary figure 3). As a result, mixing with under-ice water and between the ice horizons was possible. This could partially explain the better relative homogeneity of DMSP δ^34^S values at station 12 (Fig. 2) and the observation that bottom/ice–ocean interface isotopic values were always very close to the surface oceanic waters range (Fig. 2). Following the same line, it is also likely that the high permeability and active brine cycling that developed in the relatively warm conditions of the AWECS study in the Western Weddell Sea might have homogenised the DMSP δ^34^S profiles towards oceanic values (Fig. 1a), as observed for other biogeochemical variables during that study^30^.

If mixing processes help to understand some of the variability in sea ice DMSP δ^34^S, other processes need to be considered to explain the origin of the most extreme isotopic values recorded. These extreme values could be the expression of fundamental differences in DMSP cycling and DMSP metabolism between the extreme physicochemical conditions of isolated brine inclusions in cold sea ice and less extreme conditions in warmer ice and ocean habitats.

Results of our controlled experiments with *F. cylindrus* cultures suggest that part of the variability in sea ice DMSP δ^34^S could originate directly from the DMSP metabolism of sympagic microalgae, driven by salinity and temperature. Apparent fractionation factors from sulfate to DMSP in cultures in brine temperature and salinity conditions were larger (between −4.3 and −4.2‰) than in cultures in cold oceanic conditions (−3.4‰). The exact mechanism driving this difference in fractionation remains uncertain. Enzymatic biotransformation of assimilated sulfate to methionine followed by methionine transaminase are the common metabolic pathways of DMSP in most oceanic taxonomic groups^32,46^. Several steps in these pathways remain poorly understood^41,42^. Depending on physiochemical conditions, reactions in these pathways could be catalysed by different enzymes isoforms, with different kinetic properties and different cellular compartments localisations^41^. Looking at the proteome response to increased brine salinity in *F. cylindrus*, Lyon et al.^23^ suggested that degradation of light harvesting proteins could be an alternative source of methionine for DMSP synthesis. They also identified key enzymes that could catalyse the conversion of methionine to DMSP, enzymes that were not identified in oceanic diatoms^42^.

The difference in fractionation calculated from our cell cultures is too small to explain all the variability in DMSP δ^34^S observed in our sea ice samples (Figs. 1a and 2a, b). The most extreme sea ice DMSP δ^34^S values were measured in conditions much colder and saline than the conditions reached in our controlled experiments (Fig. 2a, b). Larger fractionations could potentially be expected at lower temperature/higher salinities. Also, the controlled experiments targeted a single microalgal species of diatom while assemblages in natural sea ice are characterized by a wide variety of microalgal taxa. Some of the variability observed in our samples could have resulted from different fractionation factors from sulfate to DMSP between different taxa. Such differences were reported for instance from previous experiments with cell cultures of different symbiotic microalgae^33^. Microalgal cell counts revealed a large variability in major algal taxonomic groups between the different sea ice horizons during the two seasonal transitions of the YROSIAE study in the McMurdo Sound (Fig. 2a, b). While the ice–ocean interface horizons (and surface horizon during the spring–summer) were dominated by diatoms, other horizons showed larger contributions of other flagellates and important populations of dinoflagellates especially during the winter–spring transition when the largest variability in DMSP δ^34^S was detected (Fig. 2a, b). Apparent fractionation factors from sulfate to DMSP in these microalgal groups in brine conditions should be assessed to expand this hypothesis.

Finally, some of the variability in sea ice DMSP δ^34^S could potentially be explained by processes of the DMSP cycle controlled by bacteria and heterotrophic protists. Here again, these processes could be particularly relevant in the enclosed environment of the brine pockets that develop in cold ice where most of the extreme isotopic values were observed (Fig. 2a). Rates of heterotrophic production are known to overcome rates of autotrophic production in the physiochemical conditions prevailing in these pockets, including light and nutrient limitations^20^. Bacteria have recently been shown to be able to produce DMSP^2^. As discussed by Curson et al.^2^, bacteria could use other S sources than sulfate for this production, such as methylated sulfur compounds with different S isotopic signature^32^. Variability could also arise from recycling metabolic processes in the brine pockets where extremely high dissolved DMSP have been reported in the past^22^. In this enclosed environment, DMSP would be continuously turned-over, released from the algal cells as an exudate, or through cell senescence, viral lysis or grazing, then metabolised by heterotrophic protists and bacteria and remineralised. Such recycling metabolic processes could lead to S isotope fractionation, as previously shown for the cleavage of DMSP to DMS^28^. As a result, some of the S re-entering the sulfur pool available to microalgae could have a distinct S isotopic signature than the original sulfate.

Altogether, the results of this study show that S isotope measurements could refine our current ability to track specific sources of DMSP, and potentially other biogenic sulfur compounds, in the complex sea ice microbial environment. Whilst it was not possible to identify all the exact mechanisms behind the variability in sea ice DMSP δ^34^S, brine inclusions connectivity and its effect on mixing, and the cycling of DMSP by bacteria and heterotrophs in the isolated brine pockets definitely warrant some further consideration, The unusual fractionation from sulfate to DMSP observed in the microalgal cell cultures in brine conditions remain very intriguing and may point to unique metabolic pathways of assimilatory sulfate reduction and DMSP synthesis. Future experiments should explore S fractionations in different cultures of sea ice microalgal and bacterial DMSP producers in brine temperatures and salinities to develop the potential applications of the present work. Additional physiochemical control parameters such as light and nutrient supply could also be considered.

## Methods

### Sampling

Three sets of ice cores were collected for this work. The first set was sampled in the McMurdo Sound during the YROSIAE (Year Round Survey of Ocean-Sea Ice-Air Exchanges in Antarctica) time series study in 2011–2012. One fast ice station located at Cape Evans was sampled seven times throughout the seasons (YRS3, 4, 6, 8, 9, 11, and 12) as described in ref. ^13^. The second set was sampled in the Western Weddell Sea during the Antarctic Winter Ecosystem and Climate Study (AWECS/ANT-XXIX/6) on the RV-Polarstern in June–August 2013. A general overview of the AWECS sampling strategy and area is given in ref. ^30^. Three pack ice stations of AWECS were analysed in this work (AW493, 500, and 506B). The last set of ice cores was collected during the PIPERS (Polynyas and Ice Production in the Ross Sea) study on the RV Nathaniel B. Palmer in the Ross Sea in April–June 2017. Two stations were visited in the marginal ice zone (PIP1 and 4), and two in the central Ross Sea ice pack (PIP29 and 23).

The same ice coring procedure^13^ was followed during the three studies. Briefly, an electropolished stainless-steel (SS) ice corer with an internal diameter of 14 cm was used to retrieve ice cores. Cores were immediately wrapped in polyethylene (PE) bags and kept at <−30 °C horizontally in the dark to prevent brine drainage from the cores and limit the physiological activity of ice algae^47^. One core per sampling event was fully dedicated to all the S analysis (DMSP concentrations and S isotopes, sulfate concentrations and S isotopes). Two other cores (physical and biological cores) were used to determine ancillary parameters as described below. The distance between the three cores collected was always <20 cm to limit spatial variability.

Liquid samples (sea water and brine) were collected during the PIPERS study. Sea water was sampled at three different SOCCOM float locations in the Ross Sea sector of the Southern Ocean using a CTD-Rosette sampler (5 m depth). Liquid samples were transferred to pre-acidified no headspace glass vials for long-term conservation at 4 °C in the dark^48^.

### DMSP and sulfate concentrations and δ^34^S analysis

The S dedicated ice core was cut into 5 or 10 cm vertical sections. Several smaller ice cuboids were then cut in the centre part of these sections for the different S analysis. Ice cuboids for DMSP concentrations were cut and processed in the field within 48 h of sampling. DMS was first extracted from the ice matrix using the dry crushing technique^47^. This technique was developed to prevent artificial conversion of DMSP to DMS on melting. One of the ice cuboids was inserted with two grade-316 SS marbles into an air-tight grade-316 SS container. The container was mechanically shaken by a fast up and down motion of the crushing device, reducing the cuboid into a fine ice powder by repeated impacts with the marbles. The ice powder resulting from the crushing step was retrieved and the DMSP content was then quantified as DMS after cold alkali cleavage of DMSP into DMS. The ice powder was left to melt at 4 °C overnight with NaOH pellets in several sealed sparging vials equipped with a Teflon septum. One vial was then connected to a traditional purge-and-trap apparatus, and DMSP quantified as DMS with an Agilent 7890A GC-dual FPD system (DB1-Sulfur Specific column)^13^. The system was calibrated with pure (>99%) DMS (Merck®) dilutions in Milli-Q water. Triplicate measurements of DMSP in ice samples gave relative standard deviations <12%.

Ice cuboids for sulfate concentrations and sulfur isotope measurements were cut in the S dedicated ice core sections in the home laboratory. Ice core sections were kept at all time wrapped in PE bags in the dark and at −30 °C between sampling and analysis. This storing procedure has been proven effective in limiting brine drainage from the samples and physiological activity of microalgae^47^. Storage times were 4 years, 3 years, and 5 months for the YROSIAE, AWECS, and PIPERS ice cores sets, respectively. Total DMSP concentrations are usually well preserved after long-term storage in ice cores as shown by the storage tests (>2 years) of Stefels et al.^47^.

Sulfur isotope measurements are presented in this study using the standard delta (δ) notation (δ^34^S) and reported relative to the international reference standard Vienna Cañon Diablo Troilite (V-CDT) in units of permil (‰) following equation:

$$\delta ^{34}S = \left( {\,^{34}R_{\mathrm{sample}}/^{34}R_{\mathrm{std}}} \right) - 1$$

where ^34^R is the integrated ^34^S/^32^S ion-current ratio of the sample and standard peaks. A sparging vial was connected to a purge-and-trap apparatus (see ref. ^27^ for a detailed description), and DMS resulting from the base-cleavage of DMSP subsequently transferred via a six-way valve (Valco Instrument Co, TX, USA; heated to 80 °C) into a Trace GC (Thermo, Germany) for separation to individual compounds using a Agilent J&W capillary column (DB-1, 60 m × 0.32 mm ID × 1.0 μm). Separated DMS was then transferred via a heated (200 °C) transfer line to a multi-collector inductively coupled plasma mass spectrometer (GC-MC-ICPMS, Neptune *Plus*, ThermoFischer, Germany) to determine its sulfur isotopic composition. Standard DMS and DMSP solutions with known δ^34^S values (−3.0‰ and 6.2‰, respectively) were injected for calibration every four samples, and a bracketing technique was applied to correct for instrumental mass bias^27,49^. Analytical precision and accuracy of DMS and DMSP analysis of standards were usually better than 0.2‰ (1*σ* standard deviation). The precision of sulfur isotope analysis for duplicate or triplicate samples of seawater or ice averaged 0.3‰ but in few extreme cases reached up to 1‰.

Quantification of sulfate was determined from a melted ice cuboid. The melt aliquot was filtered with polycarbonate filters (0.45 μm) to remove particulate organic matter. Aliquot (50 μL) of the filtered solution was diluted 400× and introduced in a Dionex-ICS5000 liquid chromatograph for ion analysis ([SO_4_^2−^]) precision of these analyses were around 20 nM. Split of this solution was treated with 10% BaCl_2_ solution and the BaSO_4_ analysed for its sulfur isotope composition by a conventional elemental analyser (EA) coupled to isotope ratio mass spectrometer (IRMS) method^50^ using Delta Plus, Thermo. The sulfur isotope reference materials NBS-127 (BaSO_4_; δ ^34^S = 21.1‰), IAEA-S-1 (Ag_2_S; −0.3‰), and IAEA-SO-6 (BaSO_4_; −34.1‰) were purchased from the National Institute of Standards and Technology (NIST) and used for calibration. Precision of this method for duplicates/triplicates was usually better than 0.3‰.

Samples on gas chromatographs and mass spectrometers were always run in random sequences. Samples were mixed from different field studies and sampling depths. Blanks and standards were also introduced between the different runs.

### Ancillary parameters

Sea ice temperature *T* (°C) was always measured in situ with a fast-response handheld portable digital thermometer equipped with a calibrated probe (TESTO®720)^51^. The probe was inserted in 4 mm diameter holes drilled to the centre of the physical core at 5 cm intervals. The precision of the probe was ± 0.1 °C with an accuracy of ± 0.2 °C. Brine salinity (*Sb*) was directly computed from sea ice temperature (*T*) assuming thermodynamic equilibrium of the brine with surrounding ice^52^:

$$Sb = \left( {1 - \frac{{54.11}}{T}} \right)^{ - 1}.1000$$

Brine volume fraction (*Vb*) (brine volume/bulk ice volume) was computed from sea ice temperature (*T*) and bulk ice salinity (*S*) using the equations of Cox and Weeks^52^ for ice temperature <−2 °C and of Lepparänta and Manninen for ice temperature ≥−2 °C^53^.

Microalgal taxonomy was determined on the biology core through light microscopic cell enumeration in 200 mL aliquots of melted sea ice stored in brown glass bottles and preserved with acid Lugol’s solution. The bottles were stored in the dark until the cells were settled from a 50, 10, 2, or 1 mL volume for up to 24 h^54^. Then, the settled cells were visualized (measured and enumerated) using a Leica Leitz DM IL inverted light microscope equipped with 10× and 40× objectives and 10× and 12.5× oculars (magnification of 500×) with an attached digital camera (LeicaDC300F) for documentation. Species identification was based on Medlin and Priddle^55^, Thomas^56^, and Scott and Marchant^57^.

### Cell cultures

Cultures of *F. cylindrus* were maintained in exponential growth at 4 °C and a salinity of 34 g kg^−1^ under a 16:8 light:dark cycle (75 μE m^−2^ s^−1^) in a refrigerated incubator (RUMED® Rubarth Apparate GmbH). *F. cylindrus* was grown in artificial seawater, created by mixing filtered (0.2 μm) Antarctic seawater from the McMurdo Sound area with artificial sea salts (Instant Ocean®) to a salinity of 100 g kg^−1^ (initial salt mixture), and diluting it with mQ water to a salinity of 34 g kg^−1^. This mixing was done because the volume of Antarctic seawater available was relatively limited. A standard f/2 medium was also added to the water^58^. These culture conditions are referred in the study as cold oceanic conditions.

In the experiment, *F. cylindrus* was initially grown under cold oceanic conditions (4 °C, 34 g kg^−1^ of salinity) in 1 L Nalgene® bottles (inoculation: 5·10^6^ cells mL^−1^). After 21 days, this control culture was sampled to determine the cell density, the carbon biomass, and the concentration in chlorophyll *a*, and DMSP (following the same purge and trap-GC procedure detailed above). The remaining volume of the 1 L culture was divided into sub-cultures (cultures in brine conditions). The salinity of both sub-cultures was modified by three additions (time 0, 4 h, 8 h) of the initial salt mixture (salinity of 100 g kg^−1^) to reach a salinity of 75 g kg^−1^ (each addition shifted the salinity by 14 salinity units). The temperature of the second sub-culture was simultaneously decreased to −4.4 °C by inserting the culture bottles in a cooling alcohol bath (PolyScience®). The cultures were then sampled after 24 h, 48 h, 72 h, and 1 week following the final salt addition and analysed for the same parameters as the control culture. Each culture (control and brine conditions) was treated with a set of antibiotics (penicillin-G and streptomycin)^59^ to limit bacterial contamination.

The sulfur isotopic composition of DMSP in each culture (control and brine conditions) was determined by GC-MC-ICPMS as described above. Artificial sea salts additions to the cultures logically modified the isotopic signature of the source sulfate for DMSP synthesis. Since all culture waters (maintenance, control, and brine conditions) were prepared from the same initial salt mixture (McMurdo Sound seawater + Instant Ocean® salts), this source sulfate had the same δ^34^S value in all the culture samples (control and brine conditions) as verified by EA-IRMS analysis of each solution (+6.5‰ ± 0.1‰). This allowed the determination of difference fractionation factors between sulfate and DMSP.

## Electronic supplementary material

Supplementary Information

## Data Availability

The datasets generated during the current study are available on the public repository Figshare^60^ under 10.6084/m9.figshare.7034975 following this link https://figshare.com/s/0d1b7b52d2d8e2ab926b.
