# Supplementary material for: Variability in sulfur isotope composition suggests unique dimethylsulfoniopropionate cycling and microalgae metabolism in Antarctic sea ice
Source: Commun Biol. 2018 Dec 4;1:212. doi: 10.1038/s42003-018-0228-y (PMC6279776; doi:10.1038/s42003-018-0228-y)

Supplementary figures

Supplementary Figure 1

Depth profiles of sea ice DMSP concentrations, DMSP δ^{34}S values, SO_4^{2-} concentrations and SO_4^{2-} δ^{34}S values for the 4 stations of the YROSIAE study in the McMurdo Sound. SO_4^{2-} concentrations measured in sea ice were weighted by the brine volume fraction to illustrate concentrations available to microalgae in the brine habitat. The vertical dashed-line indicates the mean δ^{34}S of oceanic SO_4^{2-}.

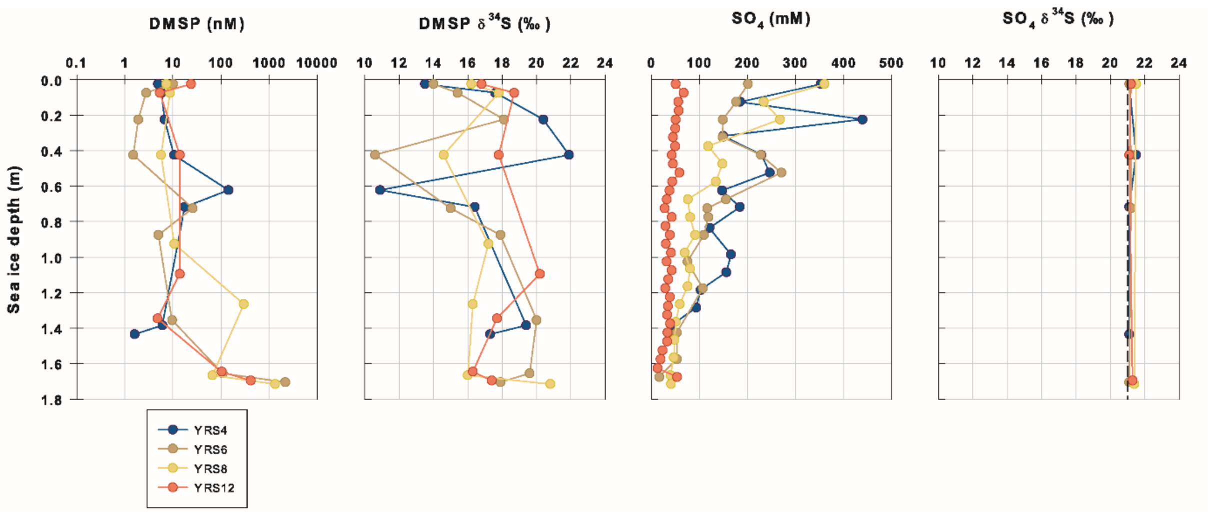

Supplementary Figure 2

Depth profiles of sea ice DMSP concentrations and DMSP δ^{34}S values for the 4 stations of the Pipers study in the Ross Sea, and the 3 stations of the AWECS study in the Western Weddell Sea.

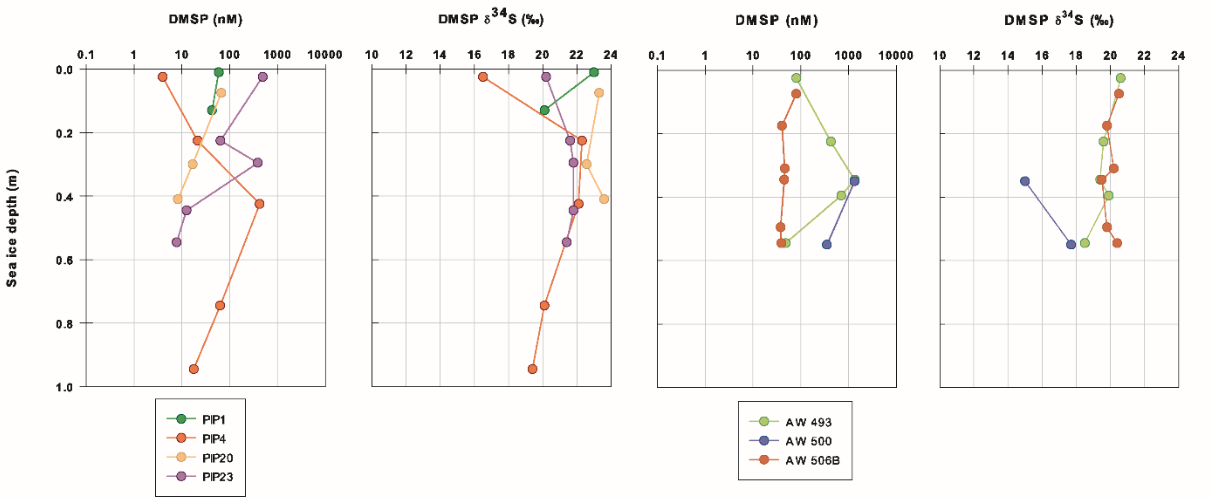

Supplementary Figure 3

Depth profiles of sea ice brine volume fraction (%) for the 4 stations of the Yrosiae and Pipers study in the McMurdo Sound and Ross Sea, and the 3 stations of the AWECS study in the Western Weddell Sea. The vertical dashed line corresponds to the theoretical brine volume fraction threshold (5%) below which sea ice is considered as impermeable to fluid transport.

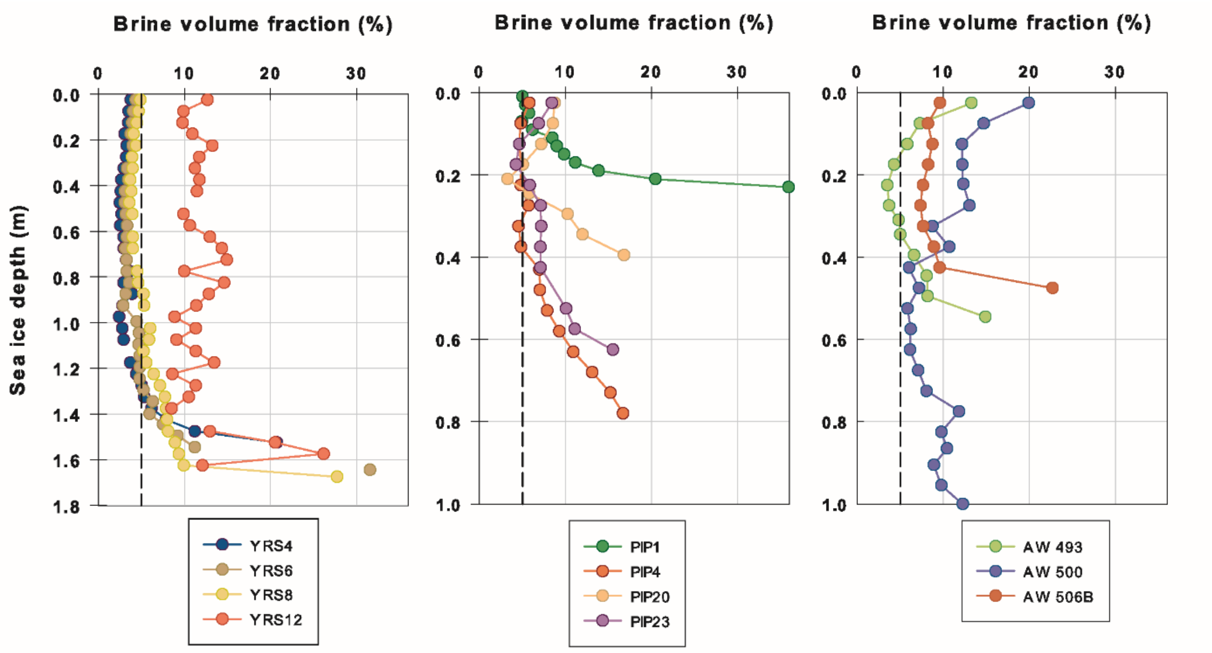

Supplementary Figure 4

DMSP $\delta^{34}S$ values vs. brine volume fraction (%) for the 4 stations of the Yrosiae study in the McMurdo Sound. The vertical dashed line corresponds to the theoretical brine volume fraction threshold (5%) below which sea ice is considered as impermeable to fluid transport. The horizontal dashed lines represent the range of DMSP $\delta^{34}S$ values observed in surface oceanic waters.

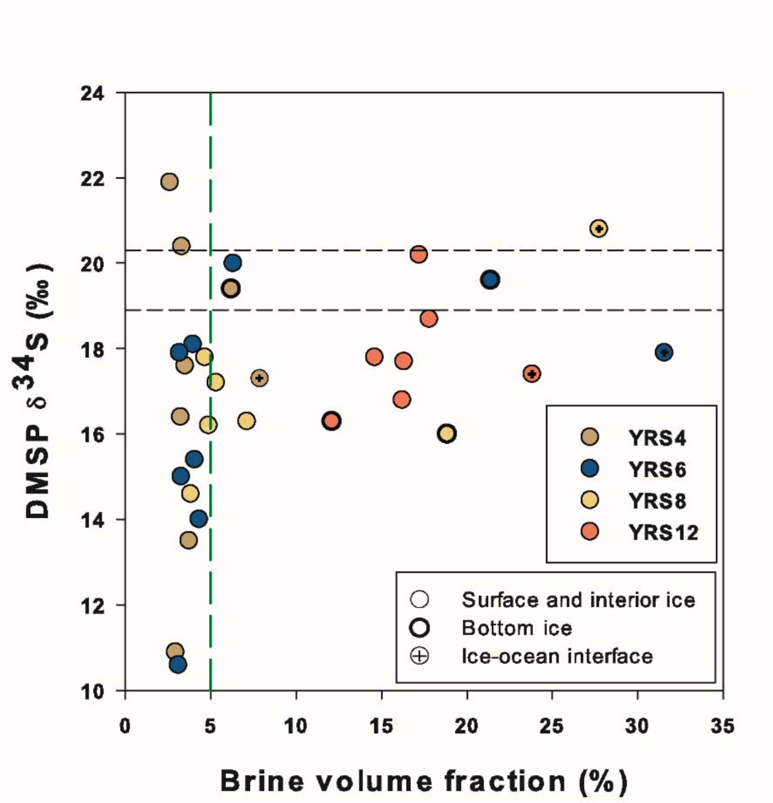

Supplement: Supplementary file 1 — Supplementary Information [file 42003_2018_228_MOESM1_ESM.pdf]
